# Biomarkers and their place in dementia research and practice‐ the lancet commission recommendations

**DOI:** 10.1002/alz.085065

**Published:** 2025-01-09

**Authors:** Lon S. S. Schneider

**Affiliations:** ^1^ Keck School of Medicine of USC, Los Angeles, CA USA

## Abstract

**Background:**

Research on biomarkers for Alzheimer’s pathology has progressed rapidly. We summarize the evidence and make recommendations about biomarkers for future clinical use.

**Method:**

Our interdisciplinary, international, multicultural group of experts in the Lancet Commission on dementia adopted a triangulation framework, prioritizing systematic reviews and meta‐analyses and agreed on the best evidence for recommendations.

**Result:**

Amyloid prevalence increases with age (>20% age 70; >30% age 80). As amyloid accumulation occurs many years before neurodegeneration or symptoms a positive biomarker *alone* does not strongly predict future impairment. Most amyloid positive, cognitively normal people do not develop Alzheimer’s dementia (Lopez et al 2024).

Neurodegeneration is more closely associated with cognitive decline than amyloid and tau fluid biomarkers. In a community‐based autopsy cohort, 8% of patients with plaques and tangles had incident dementia during their previous 5 years. But 68% of those with additional neurodegeneration had incident dementia. One population study of 2323 people found knowing blood‐based biomarkers status led to a tiny rise in concordance of prediction (0.88 to 0.9) in people with subjective cognitive impairment or MCI followed for 5 years. Biomarkers may not add value to prediction of Alzheimer’s dementia.

Plasma p‐tau181, p‐tau217, and p‐tau231 have as good or better accuracy than plasma Aβ42/Aβ40 ratio in predicting positive amyloid‐PET and in identifying people with amyloid pathology. Blood‐based biomarkers may be especially useful in the future to identify people with dementia who have very low likelihood for AD pathology and need further investigation.

Most research has been in samples of convenience and white populations with unclear generalisability. However, a recent community‐based sample of adults found no ethnic difference in plasma AD biomarkers when other risk factors were considered.

**Conclusion:**

For people with dementia, plasma biomarkers may overcome the challenges of PET even as their predictive accuracy needs improvement. Clinical use, however, is limited to assessing the cause in people with dementia. Amyloid plaque‐predictive biomarkers are at the point where they could be used clinically for people with dementia but minding the populations and regions where they have been validated.

